# Tunable Response of Silica–Gold Nanoparticles for Improved Efficiency in Photothermal Therapy

**DOI:** 10.3390/nano16040269

**Published:** 2026-02-18

**Authors:** José Rafael Motilla-Montes, Rosa Isela Ruvalcaba-Ontiveros, José Guadalupe Murillo-Ramírez, José Antonio Medina-Vázquez, Hilda Esperanza Esparza-Ponce

**Affiliations:** 1 Centro de Investigación en Materiales Avanzados, S. C. Miguel de Cervantes 120, Complejo Industrial Chihuahua, Chihuahua 31136, Mexico; jose.motilla@cimav.edu.mx (J.R.M.-M.); jose.murillo@cimav.edu.mx (J.G.M.-R.); 2 Facultad de Ciencias Químicas, Universidad Autónoma de Chihuahua, Chihuahua 31125, Mexico; rosa.ruvalcaba@cimav.edu.mx; 3 Facultad de Ingeniería, Universidad Autónoma de Chihuahua, Chihuahua 31125, Mexico; jmedinav@uach.mx

**Keywords:** photothermal therapy, silica nanoparticles, gold nanoparticles, localized surface plasmon resonance, core–shell nanoparticles

## Abstract

Photothermal therapy (PTT) is an emerging minimally invasive approach for cancer treatment that relies on photothermal agents capable of efficiently converting near-infrared (NIR) light into localized heat. In this work, silica–gold nanostructures (SGNs) were synthesized and systematically evaluated to investigate how silica core size influences the photothermal response of the SGNs and optimize their performance as a photothermal agent. SGNs were synthesized with silica cores ranging from 54 to 244 nm in diameter and coated with gold nanoparticles of 4–10 nm in size, enabling controlled tuning of their localized surface plasmon resonance within the NIR region. The morphology and composition were characterized by SEM, TEM, and EDS; optical properties were analyzed by UV-Vis spectroscopy. The SGNs photothermal response low-power laser irradiation at 852 nm and 1310 nm and temperature changes were monitored using a thermographic camera. A maximum temperature increase of 7.1 °C was observed for SGNs with a silica core diameter of approximately 77 nm under the 852 nm laser irradiation. Numerical simulations of the absorption efficiency showed good agreement with experimental UV–Vis spectra and thermal measurements, revealing a size-dependent shift of the absorption toward longer wavelengths for larger nanostructures. These results demonstrate that the photothermal response of silica–gold nanostructures can be rationally tuned through the control of core size and gold growth parameters, providing a framework for the design of wavelength-matched photothermal agents for PTT applications.

## 1. Introduction

Gold nanoparticles (AuNPs) have attracted significant research attention in recent years. They are used in catalysis [1], electricity generation [2], optics [3], and medicine [4,5]. Silica and gold nanoparticles are among the most accepted materials for biomedical applications. Interest in gold nanoparticles stems from their electrical, optical, and thermal properties [6], which enable a wide range of applications. In biomaterials, efforts are underway to use gold nanoparticles as alternatives to current diagnostic and therapeutic methods for many diseases. There is consensus on the safety of AuNPs, which are considered non-toxic in most cases [7] due to their chemically inert nature [8] and can be eliminated from the body after 24 h [9]. Arrangements incorporating gold nanomaterials (AuNMs) generally exhibit higher biocompatibility [10]. Amorphous silica nanoparticles are also under investigation for biomedical applications due to their reported non-carcinogenic nature [11], biocompatibility [12], established elimination routes [13], facile surface functionalization chemistry [14], and good reproducibility [13].

Cancer has attracted researchers’ attention for alternative treatments due to its rising incidence, variety, and severity [15]. Nanoparticles offer a modern and attractive alternative for cancer treatment. Nanoparticle-based therapies aim to target specific tissues to reduce side effects and reduce the amount of drug, material, or radiation required to achieve effects similar to those of traditional treatments [16,17]. These two strategies help nanoparticles lower the overall impact on the patient’s health.

Among the current cancer treatments, radiation therapy is frequently recommended for patients who are not eligible for surgery. The main drawback of radiation therapy is the possibility that recurrent tumors may develop dangerous traits [18]. Photothermal therapy (PTT) emerges as an alternative to radiation therapy. It offers improved spatial resolution and enables localized heating. This confines to a smaller area and enhances treatment specificity. The minimally invasive nature of PTT makes it a viable alternative for cancer treatment [19]. PTT involves a photothermal agent, generally metal nanoparticles such as gold nanoparticles [20], that is irradiated with a laser. The photothermal agent interacts with the laser, converting light into heat and increasing the temperature of the surrounding media. Heating biological environments, such as human tissue, to elevated temperatures causes protein denaturation, cell membrane disruption, and DNA damage, leading to cell death. Depending on the temperature increase, photothermal therapy can induce apoptosis or cause necrosis, the latter being the highest disruption level [21]. The laser wavelength used for PTT applications must be within a range that minimizes absorption and scattering in human tissue. This enables deeper penetration [22].

Gold nanoparticles convert light to heat via localized surface plasmon resonance (LSPR), a phenomenon that occurs in metallic or semiconductor nanomaterials and alters their optical, thermal, and other physical properties [21]. LSPR is a non-propagating excitation from the incident light’s electric field that moves free electrons coherently in a metallic nanomaterial’s conduction band [23]. Due to their small size, nanomaterials have free electrons that easily polarize in visible (400–700 nm) or near-infrared (700–1350 nm) radiation [24]. Nanomaterial size is a key factor in LSPR. Adjusting nanoparticle size and shape tunes LSPR and changes absorption and other properties [25,26].

PTT implementation requires highly efficient photothermal agents to convert laser radiation into heat [24]. Low conversion rates increase the risk of damaging healthy tissue and reduce treatment specificity. Developing new structures that better capture and convert radiation addresses this key challenge. A broader range of near-infrared wavelengths is also desired, as lower tissue absorption is low enough to be able to penetrate the tissue effectively [27] and determine the optimal range to achieve maximum tissue penetration while simultaneously reaching the maximum absorption of new photothermal agents.

AuNP morphologies have been explored in several studies for their potential use in PTT. The most frequently used morphologies for PTT are spheres, stars, bars, and core-shells [28]. Among gold nanostructures, core–shell structures are currently under investigation. These consist of particles composed of two or more materials, in which one material (the shell) encapsulates another (the core) [29]. Other nanostructures for photothermal therapy have been synthesized, i.e., using nanorods to build vesicles [30] and building erbium core–shell nanostructures [31]; textiles have also been functionalized with mesoporous silica [32].

A key benefit of the core–shell nanostructures is the combination of two or more materials properties, improving the functionality of the composite. Using a silica core and a gold shell for phototherapy prevents AuNPs agglomeration because AuNPs are anchored to the silica core [33]. As the AuNP optical properties depend on this attachment, it helps maintain the material’s optical response and functionality in the human body. Additionally, the synthesis of the silica core is relatively easy to do, the nanoparticles’ morphology and size are easy to modify, and most SNP synthesis methods are reproducible [13]. The above-mentioned properties of the gold and silica nanoparticles allow a simple, modifiable, and reproducible way to design a material for photothermal therapy.

In a previous study involving silica–gold nanostructures with an average particle size of approximately 132 nm, a temperature increase above 4 °C was reported under near-infrared irradiation [34]. Building on this work, the present study aims to investigate how the optical and photothermal response of core–shell silica–gold nanostructures (SGNs) can be modulated through controlled variation of the silica core size and gold growth parameters.

SGNs with different diameters were synthesized, coated with gold nanoparticles, and their photothermal response was evaluated under irradiation at two near-infrared wavelengths to analyze the dependency of the core size and irradiation wavelength on the photothermal response. The results demonstrate that comparable photothermal responses can be achieved either by reducing nanoparticle size and matching the absorption to shorter wavelengths, or by using larger nanoparticles and irradiating them with longer wavelengths at lower laser power. The SGNs tunability highlights the potential of these structures as adaptable photothermal agents and provides a framework for optimizing their performance in photothermal therapy applications.

## 2. Materials and Methods

### 2.1. Synthesis of Silica Nanoparticles Decorated with Gold (SGNs)

The synthesis of silica nanoparticles decorated with gold (SGNs) involves four steps: (1) synthesis of silica nanoparticles (SNPs), (2) functionalization (SFNPs), (3) synthesis and attachment of ultrafine gold particles to the surface of the SNPs (SAuNPs), and (4) the growing of gold seeds to form larger gold nanoparticles (SGNs), allowing adjustment of the nanoparticle absorbance within the therapeutic window.

The SNPs were prepared using a modified Stöber method. Deionized water and Ethanol (JT. Baker, Phillipsburg, NJ, USA) were mixed, and the reaction was carried out in a water bath at 50 °C. Once the desired temperature was reached, tetraethyl orthosilicate (TEOS, Aldrich, St. Louis, MO, USA) was added to the solution, and the reaction was maintained for 90 min. After completion, the particles were washed three times with ethanol.

This procedure was repeated under different experimental conditions to obtain SNPs with varying diameters. The synthesis conditions and the resulting SNPs sizes are summarized in Table 1.

The SNPs were subsequently functionalized to promote electrostatic interactions between the gold nanoparticles and the SNPs’ surfaces. An ethanol–water mixture with a 3:1 ratio was used to prepare a 12 mM solution of APTES (Sigma-Aldrich, St. Louis, MO, USA). Then, 10 mL of the 12 mM APTES solution and 10 mL of the 10 mg/mL SNPs solution were diluted in 20 mL of ethanol. The resulting solution was then placed in a water bath at 70 °C with constant magnetic agitation for 3 h. After functionalization, the particles were washed three times and redispersed three times in deionized water.

The next step involved the formation of nucleation sites for gold growth. Ultrafine gold particles (<1 nm) were synthesized and attached to the functionalized silica nanoparticles to obtain SAuNPs. To prepare the gold seeds, 25 mL of HAuCl_4_ 25 mM (Sigma-Aldrich, St. Louis, MO, USA) was added to 20 mL of deionized water, then the pH was adjusted to 7 with NaOH 0.1 M (Sigma-Aldrich, St. Louis, Mo, USA). The gold seeds could be used immediately for seed-mediated growth or stored at 3 °C in the absence of light to prevent agglomeration. To obtain the SAuNPs, the gold seeds were mixed with the SFNPs and stirred in a water bath at 95 °C for 30 min. After the reaction, the particles were washed and redispersed three times in deionized water.

For the shell growth process, a gold hydroxide solution was prepared by mixing 200 mL of deionized water with 0.6 g of K_2_CO_3_ (Jalmek, NL, MX) and 10 mL of HAuCl_4_, (25 mM). This solution was left to stand overnight at room temperature to facilitate the formation of Au(OH)_4_^−^ ions. To grow the gold nanoparticles, the SAuNPs were mixed with the gold hydroxide solution and reduced using NaBH_4_ 6.6 mM (Sigma-Aldrich, St. Louis, MO, USA), yielding the final SGNs. Finally, the resulting solution was centrifuged, washed, and dispersed in deionized water.

### 2.2. Material Characterization

To analyze the morphology of SNPs, a drop of the SNP solution was sonicated for 5 min and then deposited onto a silicon substrate. Micrographs of SNPs were obtained by Field Emission Scanning Electron Microscopy (FESEM) using a JSM-7401F microscope (JEOL, Tokyo, Japan).

Similarly, to analyze the morphology of SAuNPs and SGNs, a drop of the nanoparticle solution was sonicated for 5 min, deposited onto a copper grid, left to dry, and subsequently analyzed. Micrographs and elemental chemical analysis (energy-dispersive X-ray spectroscopy—EDS) for the SAuNPs and SGNs were obtained using a Hitachi 7700 Transmission Electron Microscope (TEM). From the acquired micrographs, the average diameter of the SNPs and the average diameter of the gold nanoparticles on the surface of the SNPs were estimated using Image J software version 1.54g [35]. More than 300 particles or regions were analyzed for each sample.

The optical absorbance and localized surface plasmon resonance (LSPR) characteristics of the SAuNPs and SGNs were evaluated by recording UV–Vis absorption spectra using a Thermo Scientific Evolution 220 UV–Vis spectrophotometer (Thermo Scientific, Waltham, MA, USA).

### 2.3. Evaluation of the Thermal Effect

**Figure 1:**
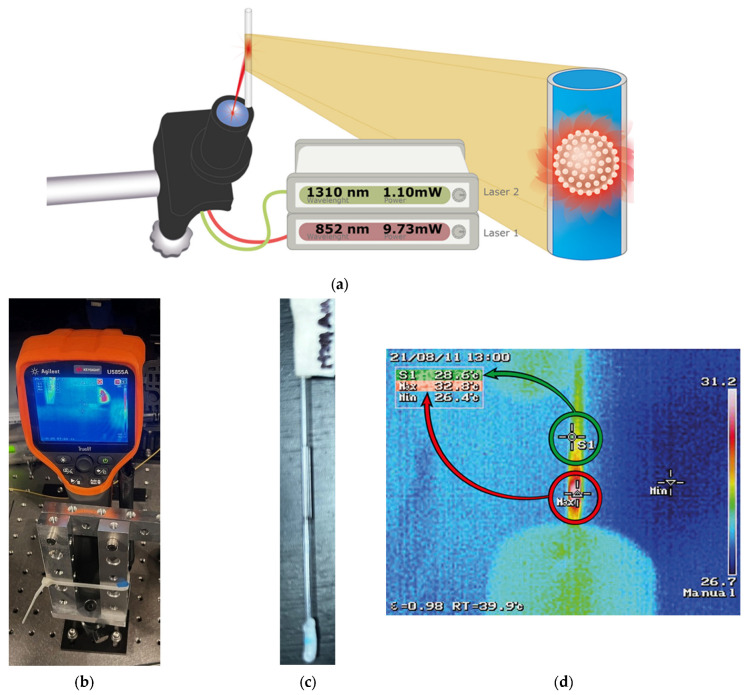
(**a**) Schematic representation of the SGNs thermal-effect evaluation system. (**b**) Experimental set up illustrating thermal imaging camera monitoring the temperature increase in a capillary tube containing SGNs. (**c**) Capillary tube with SGNs. (**d**) Thermal image showing temperature measurements at points S1 and Max.

The evaluation of the thermal effect of the SGNs was performed on an antivibration table in a particle-controlled darkroom. A multi-channel fiber-coupled laser source MCLS1 (Thorlabs, Newton, NJ, USA) was used for irradiation. The 852 nm (9.73 mW) and 1310 nm (1.10 mW) laser channels were employed, as shown in Figure 1a,b. Figure 1c shows a capillary containing SGNs dispersed in water at a concentration of 1 mg/mL, while Figure 1d shows the thermal camera screen, which records and measures the locations where the SGNs reached their highest temperatures.

The experiment was as follows: the capillary was filled with the nanoparticle solution described above. The temperature was measured at a specific point (S1) in the capillary. After 10 min of irradiation, the highest temperature inside the capillary (Max) was recorded and reported, as shown in Figure 1d.

### 2.4. Simulation of SGNs Absorption Spectra

To compare the experimental UV–Vis absorption spectra with theoretical calculations, the web-based Mie Calculator developed by the University of Information Technologies, Mechanics and Optics, was used [36].

## 3. Results and Discussion

**Figure 2:**
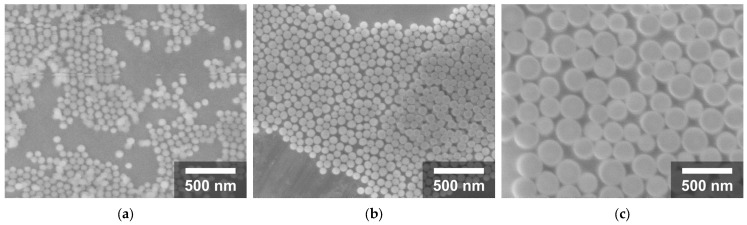
(**a**) 04 SNPs, (**b**) 05 SNPs, and (**c**) 08 SNPs. Micrographs were obtained at a nominal 50,000× magnification.

Several SNPs were synthesized under different conditions to control their size. The synthesis parameters and average diameter of the resulting nanoparticles are summarized in Table 1, and representative micrographs are shown in Figure 2.

SNPs with different diameters were successfully synthesized by modifying the concentrations of TEOS, NH_4_OH, H_2_O, and ethanol in the Stöber process. By adjusting these synthesis parameters, the resulting SNPs’ diameters ranged from 54 to 245 nm. The reactant concentrations, synthesis conditions, and corresponding average diameters of the synthesized SNPs are summarized in Table 1. The FESEM micrographs shown in Figure 2 were acquired at the same magnification and clearly illustrate the size differences among the SNPs. In all cases, the nanoparticles exhibit a spherical morphology, indicating good control over particle formation and suggesting a relatively narrow size distribution.

**Figure 3:**
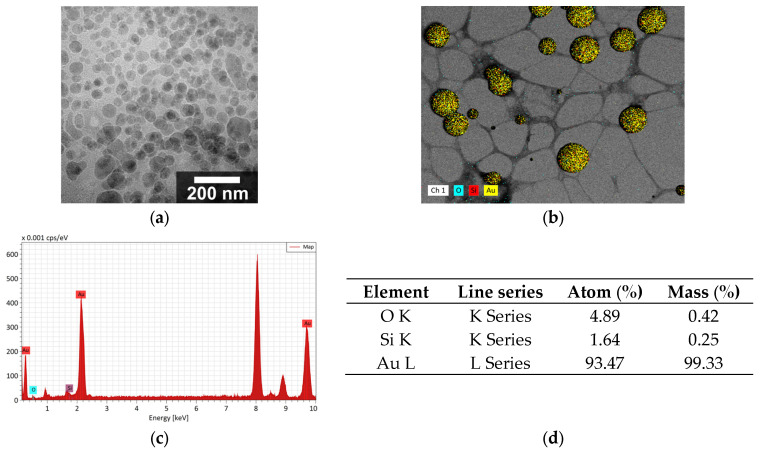
(**a**) TEM micrograph of gold nanoparticles. (**b**) Elemental mapping of AuNPs. (**c**) EDS spectra of AuNPs. (**d**) Elemental distribution obtained from EDS mapping of AuNPs.

AuNPs with diameters smaller than 5 nm were synthesized and subsequently used to decorate the surfaces of the SNPs. TEM micrographs of the SAuNPs (Figure 3a,b) confirm their nanoscale dimensions. Some degree of agglomeration was observed after deposition on the copper–carbon grid, which is commonly attributed to drying effects during sample preparation.

The seeding process resulted in AuNPs anchored to the SNPs’ surface with sizes ranging from 3 to 8 nm, and standard deviations of 0.63 to 2.86 nm. In all samples, the presence of gold and silica was confirmed by elemental chemical analysis (Figure 3c,d).

**Figure 4:**
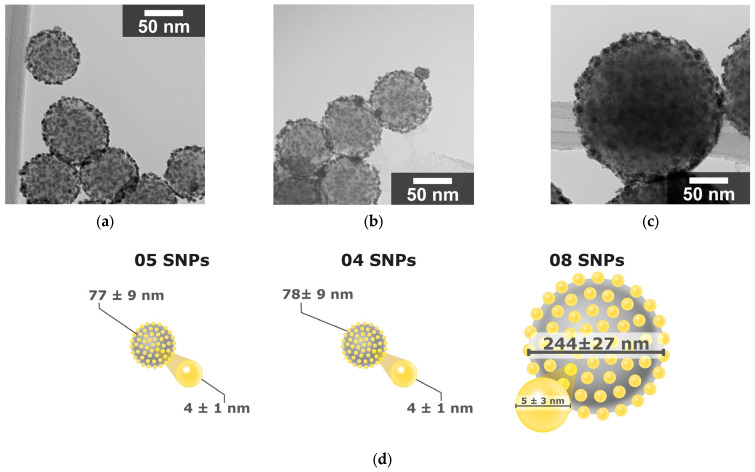
TEM micrographs of silica nanoparticles decorated with gold (SGNs): (**a**) 04 SGNs, (**b**) 05 SGNs, and (**c**) 08 SGNs. Micrographs were obtained at a nominal 100,000× magnification. (**d**) Schematic representation of the average diameters of the silica core and gold nanoparticles.

After silica functionalization and decoration with gold seeds, the SAuNPs were subjected to shell growth to increase the gold nanoparticle size. The resulting core–shell nanostructures retained the spherical morphology of the silica core, with gold nanoparticles uniformly distributed on the surface, as observed in the TEM micrographs (Figure 4a–d).

**Figure 5:**
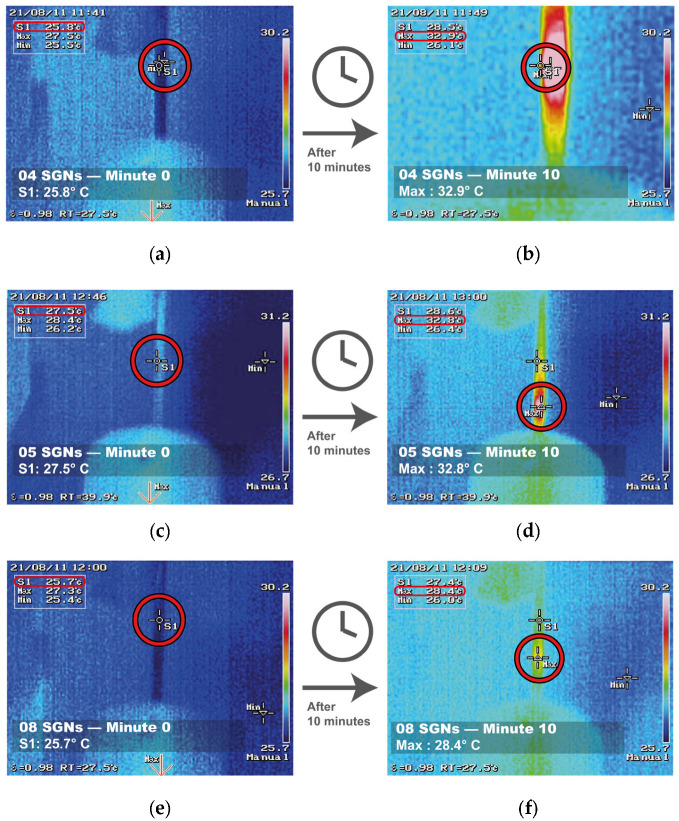
Thermal response of SGNs under near-infrared laser irradiation. The temperature of 04 SGNs was recorded at (**a**) minute 0 and (**b**) minute 10. Similarly, the temperature of 05 SGNs was registered at (**c**) minute 0 and (**d**) minute 10, and that of 08 SGNs at (**e**) minute 0 and (**f**) minute 10. In all cases, an increase in temperature was observed after laser irradiation.

To evaluate the SGNs’ photothermal response, aqueous dispersions of them were irradiated with a near-infrared laser. Figure 5 shows temperature profiles of different SGNPs under identical radiation conditions, as measured by a thermal camera. By coating SNPs of different sizes with a similar gold coverage, variations in the thermal response of the material were expected. Figure 5 shows the temperature profiles recorded for different SGN samples.

For sample 04 SGNs (Figure 5a,b), the temperature increased from an initial value of 27.5 °C to 32.9 °C after 10 min of laser irradiation. Sample 05 SGNs (Figure 5c,d) exhibited a higher photothermal response, with a temperature increase of 7.1 °C, rising from 25.8 °C to 32.9 °C over the same irradiation period. In contrast, the sample 08 SGNs (Figure 5e,f) showed a lower temperature increase of 2.7 °C, from 25.7 °C to 28.4 °C.

This size-dependent thermal behavior can be attributed to differences in the optical absorption of the SGNs, as variations in silica core diameter and gold growth modify the localized surface plasmon resonance, thereby affecting the efficiency of light absorption and heat generation.

A 7.1 °C increase from physiological temperature (36 °C) would yield a final temperature of approximately 43.1 °C. Temperature increases in the range observed for the 05 SGNs system fall within values commonly reported for hyperthermia-based photothermal applications under controlled conditions, where exposure to temperatures of approximately 42–46 °C for periods of around 10 min has been associated with cell necrosis in previous studies [37]. In the present study, the observed temperature rise serves as a comparative indicator of photothermal performance among nanostructures evaluated under identical experimental conditions, with the 7.1 °C increase achieved by the 05 SGNs system indicating its potential for photothermal applications under low-power near-infrared irradiation.

These results demonstrate that the thermal response of SGNs strongly depends on the silica core size, the extent of gold growth, and gold coverage on the nanoparticle surface. In accordance with Mie Theory and related theoretical studies on PTT applications of other materials [38], modifying the core size and shell thickness enables enhancement of the photothermal efficiency while operating under low-energy laser irradiation, which is advantageous for potential biomedical applications such as photothermal therapy.

Thermal evaluation was also performed for additional SAuNP and SGN samples. Gold coverage was similar for all nanoparticles, with the silica core size being the primary differentiating parameter. Table 2 shows the temperature increase recorded for the synthesized SAuNPs and SGNs. Nanoparticles with silica core sizes below 100 nm exhibited a thermal response when irradiated with the 852 nm laser, whereas nanoparticles with core sizes above 100 nm showed a temperature increase only when irradiated with the 1310 nm laser.

While the 05 SGNs exhibited the highest thermal response, it is necessary to note that some of the other tested nanostructures (i.e., 05 SAuNPs ΔT = 3.8 °C and 10 SAuNPs ΔT = 3.8 °C) showed a similar temperature increase for the 852 nm laser and the 1310 nm laser despite the latter operating at nearly nine times higher power (9.73 mW and 1.10 mW respectively). This observation suggests that thermal conversion efficiency could be further improved in some of the samples.

**Figure 6:**
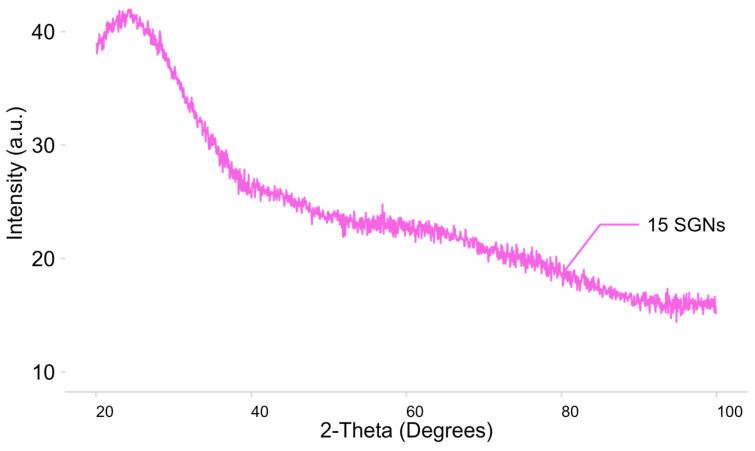
Diffractogram of 15 SGNs by X-ray diffraction.

Because crystalline SiO_2_ is associated with adverse health effects, it is essential to ensure that SNPs are amorphous for their proposed biomedical applications. The crystallinity of the SNPs was evaluated by X-ray diffraction (XRD). Figure 6 shows the X-ray diffraction pattern of SNPs, which corresponds to an amorphous material, thereby avoiding the health risks associated with crystalline silica [11,39].

**Figure 7:**
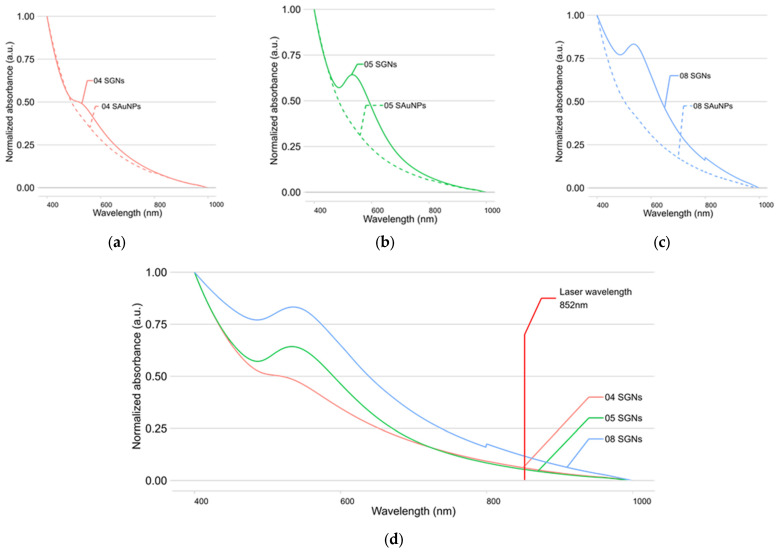
UV-Vis absorption spectra comparison of (**a**) 04 SAuNPs (dashed red) and 04 SGNs (solid red); (**b**) 05 SAuNPs (dashed green) and 05 SGNs (solid green); (**c**) 08 SAuNPs (dashed blue) and 08 SGNs (solid blue); and (**d**) 04 SGNs (solid red), 05 SGNs (solid green), and 08 SGNs (solid blue).

To relate the thermal properties of SAuNPs and SGNs to their optical properties, the UV-Vis spectra of these nanostructures are shown in Figure 7a–c. The characteristic absorbance peak of SGNs, centered around 550 nm, indicates the growth of AuNPs on the silica surface, since gold nanoparticles typically absorb in the 500–650 nm range [40]. The absence of a distinct absorption peak in some nanostructures, mainly in the SAuNPs, may be attributed to the lower amount of gold present in these samples, as a higher gold content was used for the synthesis of SGNs.

**Figure 8:**
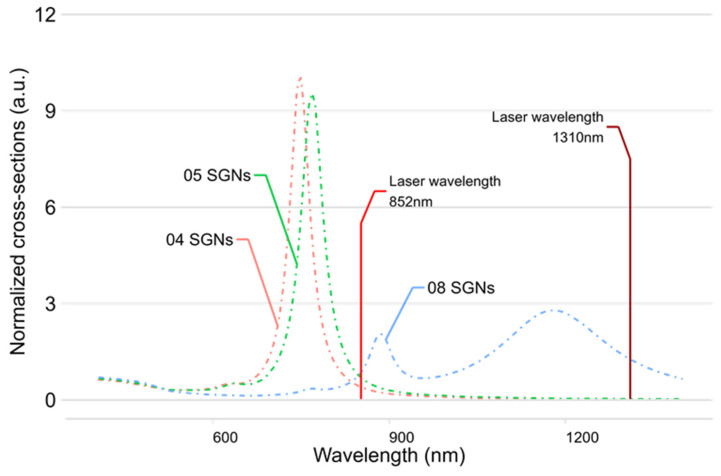
Simulated absorption efficiency Q_abs_ of: 04 SGNs (dashed red), 05 SGNs (dashed green), and 08 SGNs (dashed blue).

In addition to the UV-Vis characterization, simulations of the optical response of SGNs were performed for the synthesized nanoparticles. The silica core size and gold shell thickness obtained from SEM and TEM analyses were used as input parameters for the simulation. The simulated absorption efficiency (Q_abs_) of 04, 05, and 08 SGNs is shown in Figure 8, while for the remaining nanostructures the peak absorption wavelengths obtained from both the simulations and UV-Vis spectra are compared in Table 3.

As observed in the thermal evaluation, SGNs with sizes below 100 nm exhibit absorption peaks close to the 852 nm laser emission wavelength, whereas SGNs with sizes above 100 nm show peak absorption shifted toward the 1310 nm laser emission wavelength.

The simulated optical response of SGNs 04, 05, and 08 shows absorption at the laser emission wavelength (852 nm), which closely matches the absorption observed in the UV–Vis spectra of these nanoparticles. These samples were therefore selected for further analysis. For the remaining samples (01–10), the simulated spectra show absorption at wavelengths longer than 1000 nm, in agreement with the experimental results obtained from the thermal evaluation; the corresponding data are summarized in Table 3.

For the synthesis of SGNs, several modifications were introduced to the protocol to improve the gold coverage. It was found that the addition rate of both the gold hydroxide solution and the NaBH_4_ are critical parameters. For this study, achieving full coverage of gold nanoparticles on the surface of silica nanoparticles was systematically explored.

**Figure 9:**
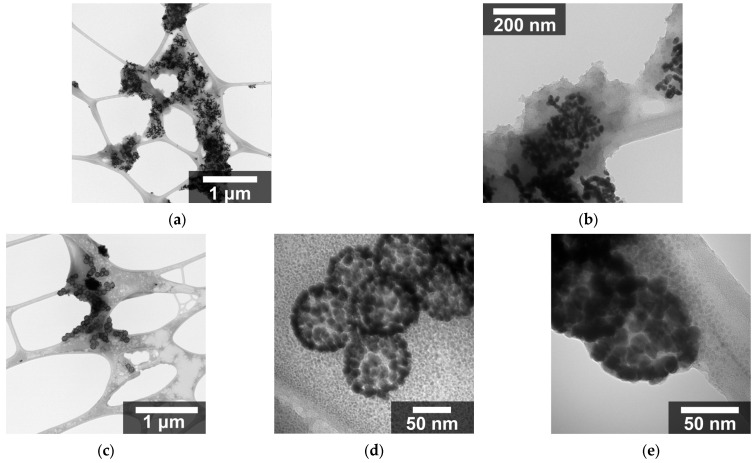
TEM micrographs of SGNs: (**a**,**b**) synthesized using four times the amount of gold hydroxide solution added in a single step, without splitting the solution; and (**c**–**e**) synthesized with the gold hydroxide solution added in a stepwise manner by dividing the total volume into four equal portions (quarters), resulting in uniform gold coverage.

Figure 9 shows the micrographs obtained when four times the amount of gold hydroxide solution was used compared to the previous synthesis, with the aim of achieving full coverage. As observed in Figure 9a,b, this approach resulted in the rupture of the silica nanoparticles. To overcome this issue, the same total amount of gold hydroxide solution was used, but it was added in a stepwise manner. Specifically, one quarter of the total volume of gold hydroxide solution was first mixed with the SAuNPs, followed by the addition of one quarter of the NaBH_4_ total volume. After allowing the reaction to proceed for a few minutes, this procedure was repeated until the entire volume of both solutions had been added.

Figure 9c–e show the nanoparticles synthesized using this modified procedure, which resulted in a uniform and complete coverage of the gold nanoparticles on the silica surface.

**Figure 10:**
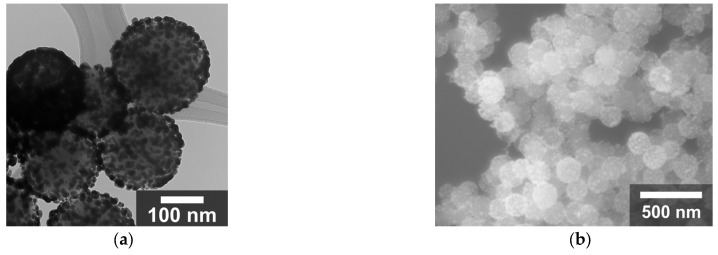
(**a**) TEM micrographs of 07 SGNs, and (**b**) FESEM micrographs of 07 SGNs.

Using this modified procedure, 07 SAuNPs were successfully synthesized, achieving good gold coverage on the surface of the nanoparticles, as shown in Figure 10.

## 4. Conclusions

In this work, a reproducible synthesis strategy was developed to obtain silica–gold nanostructures with tunable optical and photothermal properties relevant to photothermal therapy applications. By systematically varying the silica core diameter and controlling the growth of gold nanoparticles on the surface, it was demonstrated that the photothermal response of the SGNs strongly depends on nanoparticle size.

Nanostructures with silica core diameters below 100 nm exhibited photothermal heating under 852 nm laser irradiation, whereas larger particles showed a response at 1310 nm. Among the synthesized systems, SGNs with a silica core diameter of 77 ± 9 nm and partial gold coverage of 4.1 ± 1.04 nm produced the highest temperature increase (7.1 °C) under irradiation at 852 nm using a laser power of 9.73 mW. These temperature changes are intended as comparative indicators of photothermal performance among nanostructures evaluated under identical experimental conditions.

The results further highlight the importance of synthesis parameters, particularly the reagent addition rate during gold growth, in determining shell morphology and structural integrity. Stepwise addition of the gold precursor and reducing agent was shown to prevent silica rupture and promote uniform gold coverage, thereby improving photothermal performance.

Although this study does not include biological evaluation, cytotoxicity assays, or stability tests in physiological media, it establishes clear structure–property–response relationships that are essential for the rational design of silica–gold nanostructures as photothermal agents. By systematically correlating nanoparticle size, optical absorption, and photothermal heating under near-infrared irradiation, this work defines key parameters that govern photothermal performance at the materials level.

The maximum temperature increase observed for the 05 SGNs system indicates that appropriately designed silica–gold nanostructures can generate localized heating within temperature ranges commonly associated with hyperthermia-based photothermal applications under low-power irradiation. The demonstrated ability to tune the photothermal response through controlled synthesis establishes a strong materials-level foundation for subsequent biological and therapeutic evaluation.

In this context, the primary contribution of the present work lies in the development of a simple, tunable, and reproducible synthesis strategy that enables precise control over nanoparticle size and optical response, offering a versatile platform for future application-specific optimization and biological evaluation in photothermal therapy.

## Figures and Tables

**Table 1:** Synthesis conditions of SNPs and their average particle diameters measured by SEM.

Sample	TEOS (mol/L)	NH_4_OH (mol/L)	H_2_O (mol/L)	Ethanol (mL)	Temperature (°C)	Average Diameter (nm)	Standard Deviation (nm)
01	0.04	1.86	5.73	40	50	54	7
15	0.04	1.86	5.73	40	50	73	9
05	0.12	0.35	7.82	45	50	77	9
12	2	2	2	45	50	77	7
04	0.19	0.41	4.89	40	50	78	9
13	1.5	1.5	6	45	50	79	8
11	2	1.5	3	40	50	82	7
09	2	2	2	45	50	84	10
10	0.3	5.2	0	30	50	131	15
06	0.23	0.4	5.9	40	50	180	12
07	0.09	0.78	8.08	40	50	185	23
08	0.1	0.77	8.06	45	50	244	27

**Table 2:** Temperature increase as a function of particle diameter and laser wavelength for SAuNPs and SGNs.

ID	Nanostructure	Diameter (nm)	Laser Wavelength (nm)	Delta Temperature (Δ °C)
05	SGNs	77.24	852 nm	7.1
04	SGNs	77.56	852 nm	5.3
04	SAuNPs	77.56	852 nm	4.5
05	SAuNPs	77.24	852 nm	3.8
10	SAuNPs	130.97	1310 nm	3.8
06	SAuNPs	180.19	1310 nm	3.2
08	SGNs	244.34	1310 nm	2.7
06	SGNs	180.19	1310 nm	2.6
07	SAuNPs	185.44	1310 nm	2.2
08	SAuNPs	244.34	1310 nm	2.2
10	SGNs	130.97	1310 nm	1.8
01	SAuNPs	54.2	852 nm	0
01	SGNs	54.2	852 nm	0

**Table 3:** Comparison of simulated and experimental UV–Vis maximum absorption values for all synthesized nanostructures.

ID	Nanostructure	Simulation QAbs Peaks (nm)	UV-Vis Spectra Peak (nm)
01	SAuNPs	815	No peak
01	SGNs	686	549–660
04	SAuNPs	859	520
04	SGNs	899	530
05	SAuNPs	873	504–550
05	SGNs	884	535
06	SAuNPs	1419	No peak
06	SGNs	1271	533
07	SAuNPs	933, 1440	No peak
07	SGNs	833, 1087	614
08	SAuNPs	1062, 1474	No peak
08	SGNs	1021, 1421	535
10	SAuNPs	712, 947	533

## Data Availability

The data presented in this study are available from the corresponding author upon reasonable request.

## Abbreviations

The following abbreviations are used in this manuscript:

SNPs	Silica nanoparticles
SFNPs	Silica functionalized nanoparticles
SAuNPs	Silica seeded nanoparticles with gold
SGNs	Silica–gold nanostructures
PTT	Photothermal therapy
LSPR	Localized surface plasmon resonance
AuNPs	Gold nanoparticles
AuNMs	Gold nanomaterials
